# Effects of Water Stress, Defoliation and Crop Thinning on *Vitis vinifera* L. cv. Solaris Must and Wine Part II: ^1^H NMR Metabolomics

**DOI:** 10.3390/metabo12070672

**Published:** 2022-07-21

**Authors:** Violetta Aru, Andreas Paul Nittnaus, Klavs Martin Sørensen, Torben Bo Toldam-Andersen, Søren Balling Engelsen

**Affiliations:** 1 Department of Food Science, University of Copenhagen, Rolighedsvej 26, DK-1958 Frederiksberg, Denmark; kms@food.ku.dk; 2 Department of Plant and Environmental Sciences, University of Copenhagen, Højbakkegård Alle 13, DK-2630 Taastrup, Denmark; a.nittnaus@gmx.at (A.P.N.); tbta@plen.ku.dk (T.B.T.-A.); 3 Instituto Superior de Agronomia, Universidade de Lisboa, Tapada da Ajuda, 1349-017 Lisboa, Portugal

**Keywords:** *Vitis vinifera*, Solaris, grapevine, water deficit, defoliation, crop thinning, ^1^H NMR, metabolomics, FT-IR, WineScan, tyrosol

## Abstract

Proton nuclear magnetic resonance (^1^H NMR) metabolomics was employed to investigate the impact of water deficit, defoliation, and crop thinning on the chemical composition of must and wines from the cool-climate white grape variety Solaris. The obtained results show that viticultural practices (defoliation and crop thinning) affected the amino acid and sugar content of Solaris must and thereby the quality of the final wine—mainly in terms of compounds normally related to fruity aroma (i.e., isopentanol), non-sugar sweetness (i.e., proline and glycerol), and alcohol content. The content of tyrosol, a natural phenolic antioxidant with a high bioavailability, was increased in the final wine by a combination of defoliation and crop thinning. The results of the metabolomics analysis performed on the must and wine samples from the water stress experiment showed that short-term water deficit significantly affected the concentration of several flavor-related compounds, including glutamate, butyrate and propanol, of the organic acids lactate and fumarate, and of the phenolic compounds caffeic acid and *p*-coumaric acid. ANOVA simultaneous component analysis showed that the effect of water deficit accounted for 11% (*p* < 0.001) and 8% (*p* < 0.001) of the variability in the metabolite concentrations in must and wines, respectively, while viticultural practices accounted for 38% (*p* < 0.001) and 30% (*p* < 0.001) of the metabolite variability in must and wines, respectively.

## 1. Introduction

Solaris is a relatively new cultivar of *Vitis vinifera* (L.), and for this reason, the number of investigations targeting viticultural and oenological aspects related to this white grape variety is quite limited (Table 1). Solaris is a hardy grapevine that thrives in cold climates, with a good resistance to downy mildew, and that, from an agronomical point of view, has a remarkable ability to tolerate and recover from short-term water deficit [1,2,3,4].

On the chemistry side, to the best of our knowledge, most research on Solaris wines has focused on the targeted profiling of volatile metabolites and phenols, sensory analysis, as well as on the characterization of the bulk oenological properties (Table 1).

In 2015, Liu et al. investigated the volatile and non-volatile composition of Solaris wines, as well as the sensory attributes [5]. The study concluded that, from a sensory point of view, Solaris grapes can produce balanced wines characterized by medium acidity, with floral and fruity notes, which are mainly attributed to acetates and ethyl esters of short-chain fatty acids. Zhang et al. studied the impact of pre-fermentation treatments on Solaris wines and showed that cold maceration enhances the apricot and apple flavors, while a short skin fermentation increases the floral flavors of rose and elderflower [6]. Recent studies have shown that, during fermentation, the use of non-*Saccharomyces* yeasts in combination with *Saccharomyces cerevisiae* can improve the sensory attributes and chemical composition of Solaris wines [9]. Inoculation with three Metschnikowia strains (one *M. chrysoperlae* and two *M. fructicola*) promoted a similar range of volatile compounds when compared to the wine produced with a single inoculation of *S. cerevisiae*, and especially *M. chrysoperlae* was closely associated with fruity, tropical fruit, and elderflower attributes [9]. In 2020, Garrido-Bañuelos et al. described the volatile and non-volatile composition of Swedish Solaris wines compared to other white wines, including Albariño (Spain), Sauvignon Blanc (France and New Zealand), Chardonnay (France), and Chenin Blanc (South Africa). When compared to other wines, Swedish Solaris wines had significantly higher amounts of ethyl propionate, ethyl 2-methylpropanoate, diethyl succinate, ethanol, succinic acid, tartaric acid, and sucrose, important contributors to wine flavor [15].

Independently of the grape variety, the aroma and flavor profile of wines primarily stems from the chemical composition of the grapes at harvest, the vinification process, aging, and storage conditions [17,18,19,20,21]. Moreover, geological characteristics, climate, and viticultural practices, including defoliation and crop thinning can “indirectly” affect wine composition [22,23,24,25]. For white wines, numerous scientific papers are available on the effect of different agronomic practices in vineyards, including biostimulant application, irrigation, defoliation, training systems, foliar fertilization, and bunch thinning on the concentration of terpenes, thiols, C13-norisoprenoids, methoxypyrazines, and nonterpenic alcohols—the most important aroma compounds in white grapes [26]. Moderate increase in cluster light exposure after leaf removal has been shown to augment the flavonol concentration in white grapes Grechetto Gentile and Trebbiano berries [27,28]—antioxidant production being part of defense mechanisms in grape berries, which is induced by UV exposure [29]. Irrigation and crop thinning have been shown to have a positive impact on both berry weight and berry number per cluster in both red and white grapes [24]. Water is the most fundamental constituent and carrier of plant metabolism, and together with carbon dioxide, it is the foundation for plant growth and development. Irrigation is thus essential to wine production in more arid regions. However, in most cool-climate productions, water is supplied by rain or snowfall and the portion that is distributed via the soil is available for the plant to use. The amount of water absorbed is highly dependent on the type of soil and its water holding capacity, as well as on the climatic conditions of site, which determine how fast the water evaporates from the ground surface [23]. Water availability may thus vary considerably and affect plants differently depending on the phenological stage, yield, and vigor levels. 

Over the last decades, chemical analysis by untargeted spectroscopy has become an essential tool for assessing wine quality, and together with multivariate data analysis methods, as done in metabolomics, it has become a powerful tool for elucidating several aspects related to viticulture practices and wine production, origin, and fraud [30]. Metabolomics is traditionally focused on the analysis of low-molecular weight metabolites (<1.5 KDa) using advanced analytical techniques, such as nuclear magnetic resonance spectroscopy (NMR) and hyphenated chromatographic methods (i.e., gas and liquid chromatography mass spectrometry, GC-MS and LC-MS) [31]. The use of a metabolomics approach in wine science has opened new opportunities to evaluate the entire grape-growing and winemaking processes from a more holistic perspective, and typically, with an aim to gauge wine quality for production optimization and ensure traceability for fraud prevention [30]. In this context, proton (^1^H) NMR spectroscopy must be emphasized for its inherent advantages: it is quantitative, it is unbiased and relatively fast, which makes it a powerful tool for the high-throughput and unbiased analysis of food and beverages, including wine [32]. NMR allows the simultaneous detection and quantification of the individual chemical components constituting the bulk metabolome of the sample under investigation (i.e., must or wine), including primary (i.e., sugars, organic acids, and individual amino acids) and secondary metabolites (i.e., flavonoids and anthocyanins). The higher sensitivity and selectivity give NMR a leading edge over, for instance, FT-IR spectroscopy, which is routinely used to determine the bulk chemistry of grape must and wine. NMR has successfully been applied for the overall chemical characterization of wines and grape-derived products [32], to study the effect of vintage [33], terroir [34], berry shading [35], geographical origin [36], adulteration/authentication [37], the impact of sulfur dioxide on the wine metabolites [38], and the alcoholic and malolactic fermentation processes [39,40].

**Figure 1:**
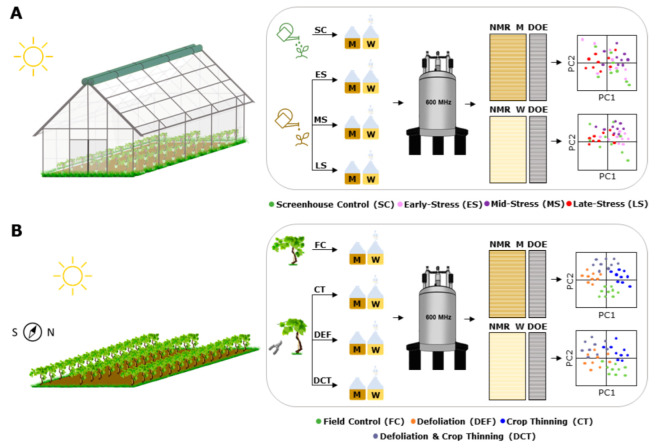
Experimental design. The water deficit experiment was carried out in an “open” screenhouse set up (**A**) where 36 pot-grown vines underwent water stress at three different phenological stages of berry development, namely pre-veraison (early-stress, ES), veraison (mid-stress, MS), and ripening (late-stress, LS). The field experiment (**B**) was performed in a north–south oriented vineyard where 32 vines underwent different viticultural treatments, namely crop thinning (CT), defoliation (DEF), and a combination of the above (DCT). Must and wine samples from all treatments were stored at −80 °C until ^1^H NMR measurements. Multivariate analysis was employed to assess the impact of different growing conditions (field vs. screenhouse), water deficit, and viticultural practices on the chemical composition of must and wine samples. M: must; W: wine; DOE: design of experiment. Color-coding for the PCA scores is given in the figure legend.

In this study, the impact of water deficit (WD), defoliation (DEF), and crop thinning (CT) on the metabolic composition of must and wine samples from Solaris grapes is investigated by ^1^H NMR metabolomics. The aims are to describe (1) the baseline metabolome of must and wines as a function of different growing conditions, (2) the changes in the bulk metabolome of must and wine samples obtained from different viticultural treatments, and (3) the changes in the bulk metabolome of must and wine samples from vines that underwent water deficit. A graphical overview of the experimental design is given in Figure 1.

## 2. Results

### 2.1. Analysis of Must and Wine Samples by ^1^H NMR

**Figure 2:**
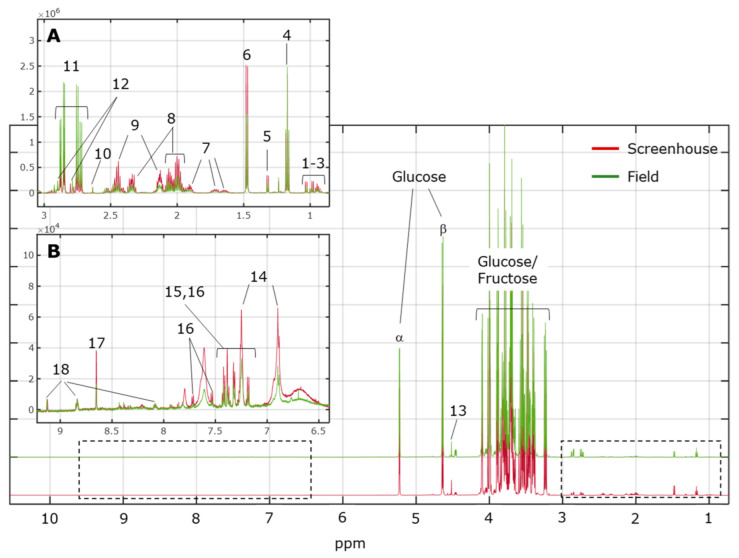
Representative ^1^H NMR spectra of must samples from the screenhouse and field experiments. (**A**) Expansion of the aliphatic region (dotted rectangle, 0.5−3.0 ppm). (**B**) Expansion of the aromatic region (dotted rectangle, 6.5−9.5 ppm). Keys: 1. isoleucine; 2. leucine; 3. valine; 4. ethanol; 5. lactate; 6. alanine; 7. arginine; 8. proline; 9. glutamine; 10. succinate; 11. malate; 12. citrate; 13. tartrate; 14. tyrosine; 15. phenylalanine; 16. tryptophan; 17. formate; 18. trigonelline.

In Figure 2, a representative ^1^H NMR spectrum of grape must from the vines grown in the open screenhouse is shown overlaid with a representative ^1^H NMR spectrum of grape must from vines grown in the field (vineyard). In the high-field region (Figure 2A; 0.50–3.00 ppm), the signals from non-aromatic amino acids (i.e., alanine, arginine, valine, isoleucine, leucine, glutamine, and proline) can be found, together with the resonances from several organic acids, including succinate, lactate, acetate, citrate, and malate. Although at very low concentrations, the typical resonance of ethanol can also be observed in the same spectral region (triplet at 1.18 ppm). At mid-field (3.01–5.50 ppm), the signals from small sugars, including glucose and fructose, resonate and, as expected, dominate the spectral landscape in all must samples. In the low-field region (Figure 2B; >5.51 ppm), the signature signals from the aromatic amino acids phenylalanine, tyrosine, and tryptophan can be found along with the alkaloid trigonelline, and formate. Metabolite assignment (level 2) is based on the yeast metabolome database (http://www.ymdb.ca/; accessed on 15 April 2022) and is given in the caption to Figure 2.

**Figure 3:**
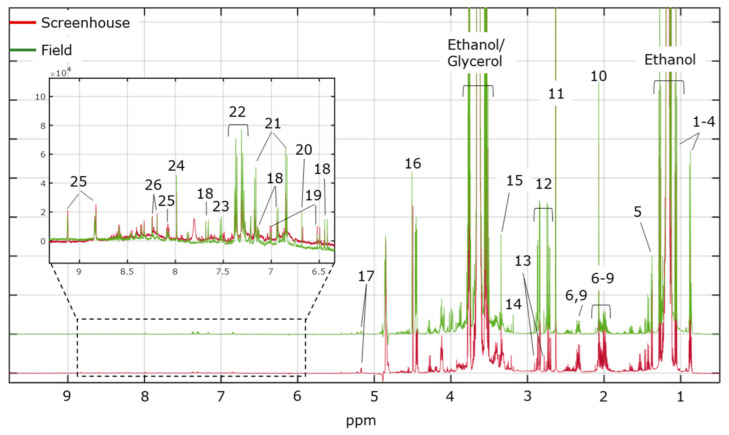
Representative ^1^H NMR spectra of wine samples from the screenhouse and field experiments. Keys: 1. isoamyl alcohol (isopentanol); 2. isobutanol; 3. butyrate; 4. 2,3-butanediol; 5. lactate; 6. glutamate; 7. glutamine; 8. methionine; 9. proline; 10. acetate; 11. succinate; 12. malate; 13. citrate; 14. choline; 15. methanol; 16. tartrate; 17. glucose; 18. caffeic acid; 19. *p*-coumaric acid; 20. fumarate; 21. tyrosol; 22. phenylethanol; 23. cytosine; 24. unknown; 25. trigonelline; 26. adenine.

Figure 3 shows representative ^1^H NMR spectra of wine samples from the screenhouse and field experiments. As expected, the ethanol signals dominate the spectral landscape followed by the intense resonances from glycerol. In the high-field region, where the methyl (−CH_3_) signal from ethanol resonates, the resonances from several organic acids can be found, including butyrate, lactate, succinate, citrate, malate, and acetate. Furthermore, the signals from shielded methyl protons from the higher alcohols isobutanol (J = 6.70 Hz, 0.90 ppm) and isopentanol (J = 6.83 Hz, 0.88 ppm) can be observed. At mid-field, the resonance from tartaric acid is found along with the complex resonances from glycerol, the methylene (-CH_2_-) ethanol signal, and residual sugars. Trigonelline, 2-phenylethanol, and tyrosol are found at low-field together with the signals from the phenolic compounds caffeic acid and *p*-coumaric acid, and the nitrogenous bases adenine and cytosine. Metabolite assignment (level 2) is based on literature data (J-coupling and chemical shift) [34,39,41,42] and the yeast metabolome database (http://www.ymdb.ca/; accessed on 15 April 2022).

### 2.2. Analysis of Wine Samples by FT-IR Spectroscopy (WineScan)

Must and wine samples were also analyzed by FT-IR spectroscopy. The reader is referred to Aru et al. (2022) for more details on the FT-IR measurements of must samples [3]. WineScan analysis of the WD wines showed no significant differences for all parameters except for malic acid, which was significantly lowered by early- and mid-stress (Table S1). As for the wine samples from the field experiment, significantly higher levels of ethanol were found in CT and DCT samples, while DEF significantly decreased ethanol compared to the control (Table S2). Glycerol fluctuations followed the variations in the ethanol content. DEF led to significantly higher levels of total acidity and tartaric acid, while CT alone led to the lowest levels. As expected, samples with the highest total acidity had the lowest pH. Volatile acidity was low in all wines, but a small increase was observed for CT wines. Concerning malic acid, DCT led to significantly higher values than CT alone. No significant differences in sugar content were observed.

### 2.3. The Must and Wine Metabolome as a Function of the Growing Condition

**Figure 4:**
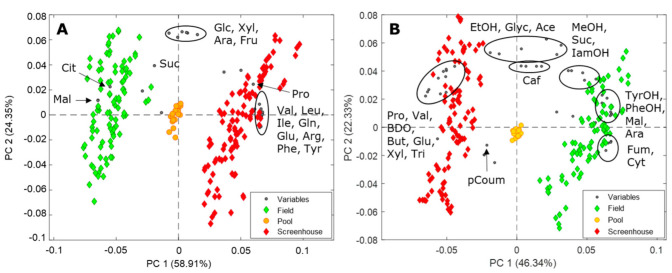
Biplots of the PCA performed on the metabolite concentrations in must (**A**) and wine (**B**) samples from the field and screenhouse trials. Keys. Ace: acetate; Ara: arabinose; Arg: arginine; BDO: 2,3−butanediol; But: butyrate; Caf: caffeic acid; Cit: citrate; Cyt: cytosine; EtOH: ethanol; Fum: fumaric acid; Fru: fructose; Glc: glucose; Gln: glutamine; Glu: glutamate; Glyc: glycerol; IamOH: isoamyl alcohol (isopentanol); Ile: isoleucine; Leu: leucine; Mal: malate; MeOH: methanol; pCoum: *p*−coumaric acid; Phe: phenylalanine; PheOH: phenylethanol; Pro: proline; Tri: trigonelline; Tyr: tyrosine; TyrOH: tyrosol; Suc: succinate; Val: valine; Xyl: xylose.

Principal component analysis (PCA) [43] was employed to obtain an overview of the variability in the metabolome of the must and wine samples. Tartaric acid was excluded from the data analysis due to inconsistency with the FT-IR measurements (see Section 3.1). Figure 4A,B show the biplots of PCA performed on the metabolite table of the must and wine samples, respectively (approximatively 83% and 68% of explained variance, respectively). Figure 4A reveals a clear metabolic difference between must samples from the screenhouse and from the field. The must samples from the screenhouse are characterized by higher levels of amino acids including valine, isoleucine, leucine, glutamine, glutamate, arginine, proline, phenylalanine, and tyrosine, while must samples from the field trial display higher levels of organic acids, including succinic acid, malic acid, and citric acid. As for the wines, Figure 4B reveals that the samples from the screenhouse display higher levels of 2,3−butanediol, proline, valine, trigonelline, and butyrate, while wine samples originating from the field display higher levels of higher alcohols, including phenylethanol and tyrosol, and of the organic acids malate and fumarate. The biplots of must and wines for both screenhouse and field experiments display a noteworthy spread along PC2, which is driven by the sugars content in must samples, and ethanol, glycerol, and acetate in the wines.

### 2.4. Impact of Water Deficit and Different Viticultural Practices on the Must and Wine Metabolome

**Figure 5:**
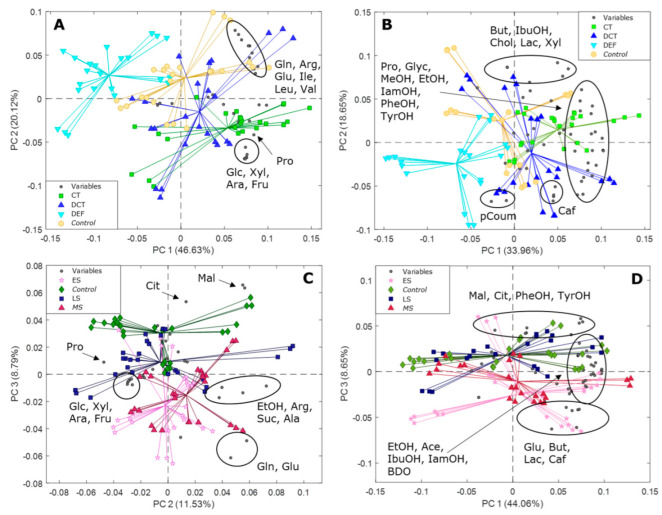
(**A**–**D**) Biplots of the PCA performed on the metabolite concentrations in must and wine samples from the field experiment ((**A**,**B**), respectively) and must and wine samples from the screenhouse experiment ((**C**,**D**), respectively). Legend: CT = crop thinning; DCT = defoliation and crop thinning; DEF = defoliation; ES = early-stress; MS = mid-stress; LS = late-stress; Variables = metabolites. Keys: Ace: acetate; Ala: alanine; Ara: arabinose; Arg: arginine; BDO: 2,3−butanediol; But: butyrate; Caf: caffeic acid; Chol: choline; Cit: citrate; EtOH: ethanol; Fru: fructose; Glc: glucose; Gln: glutamine; Glu: glutamate; Glyc: glycerol; IamOH: isoamyl alcohol; IbuOH: isobutanol; Ile: isoleucine; Lac: lactate; Leu: leucine; Mal: malate; MeOH: methanol; pCoum: *p*-coumaric acid; PheOH: phenylethanol; Pro: proline; TyrOH: tyrosol; Suc: succinate; Val: valine; Xyl: xylose.

PCA was also employed to evaluate the impact of water deficit and different viticultural practices on the metabolome of must and wine samples from the field and screenhouse experiments separately. Figure 5A,B show the PCA biplots performed on the must (A) and wine (B) ^1^H NMR datasets from the field experiment (PC1 and PC2 explain approximatively 67% and 53% of the total metabolite variation in must and wine, respectively). In Figure 5A, a clear trend is observed along PC1, where must samples from DEF and CT plants cluster in opposite PC1 quadrants. Control and DCT samples cluster in between, with the control samples close to the DEF samples, whereas the DCT samples cluster close to the CT samples. Furthermore, a weak trend is observed along PC2 with DEF and control samples clustering at positive PC2 values, while CT and DCT samples clustered in the opposite PC2 quadrant. The sample (scores) distribution along PC1 is driven by sugars and proline, which are higher in CT samples, while the amino acids glutamine, glutamate, arginine, isoleucine, leucine, and valine are higher in samples clustering at positive PC2 values. Figure 5B shows the biplot of the PCA performed on the metabolite table of the wine samples from the field experiment. The clear separation observed in the PCA biplot of the must samples (Figure 5A) is largely maintained in the PCA biplot of the wine metabolites. As before, the CT and DEF samples cluster in opposite PC1 quadrants, with control and DCT samples clustering in between. The sample distribution along PC1 is driven by proline, glycerol, methanol, ethanol, isoamyl alcohol, phenylethanol and tyrosol, which are more abundant in wines from CT treatments. 

In Figure 5C,D, the biplots of the PCA performed on the metabolite concentrations in must and wine samples from the screenhouse (water deficit) experiment are shown, respectively (PC2 and PC3 explain approximately 20% of the total metabolite variance in must, while PC1 and PC3 explain approximately 53% of the total variance in wines). In contrast to the field experiment, the high inter-sample variability is now dominating PC1 (data for must is not shown), and the impact of the water deficit experiment on the must metabolome can be observed along PC3 (approximately 9% of the metabolite variation explained, Figure 5C). Control samples cluster at positive PC3 values, while early- and mid-stressed samples cluster in the opposite PC3 quadrant. Late-stressed samples cluster in between. Sample distribution along PC3 is driven by citric acid and malic acid, which were found to have higher concentrations in control samples, and the amino acids glutamine and glutamate, highest in early- and mid-stress samples. PC2 is dominated by a high inter-sample variability, with sample distribution being driven by arginine, alanine, succinate, and ethanol (positive PC2 values), and sugars and proline (negative PC2 values).

As for the wine samples (Figure 5D), a weak stress-related trend can be observed along PC3, with control and late-stressed samples clustering at positive PC3 values, while early- and mid-stressed samples cluster in the opposite quadrant. The samples distribution is driven by malate, citrate, tyrosol, and phenylethanol, which were found to be at higher concentrations in control and late-stressed samples, while glutamate, butyrate, and lactate were higher in early- and mid-stressed wine samples. A noteworthy inter-sample variability can be observed along PC1, with samples clustering at positive PC1 values being higher in ethanol, acetate, 2,3-butanediol, isoamyl alcohol and isobutanol.

In order to scrutinize for possible additional sources of variability that potentially can be related to the observed high inter-sample diversity, PCA scores in Figure 5A–D were colored according to the pruning type (one vs. two canes for the screenhouse samples, two vs. three canes for the field samples). The results are shown in Figure S1A–D and evidence that pruning type is a major contributor to the metabolite variability in both must and wine samples from both growing conditions (field and screenhouse)—in general, the lower the number of canes, the higher is the metabolite concentration in the must and wine samples.

**Figure 6:**
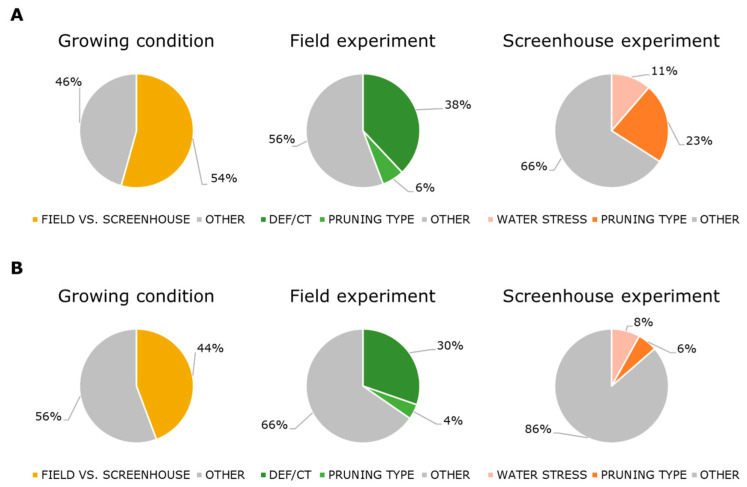
ASCA performed on the NMR metabolite concentrations from must (**A**) and wine (**B**) samples from the field and screenhouse experiments. Each experimental factor is represented with a slice in the chart. Effect sizes (%) are given next to the slices. All effects are significant (*p* < 0.001, 1000 permutations). Stress-related classes in the field (DEF/CT) experiment = control, DEF, CT, and DCT; Stress-related classes in the screenhouse (water stress) experiment = control, early-stress, mid-stress, and late-stress. Grey areas represent the residual and unknown source of variability.

ANOVA simultaneous component analysis (ASCA) [44] was employed to quantify the effect of the different growing conditions (field vs. screenhouse), treatments − namely water deficit (early-, mid-, and late-stress) and viticultural practices (DEF, CT, and DCT) − as well as the pruning type on both growing conditions (one vs. two canes for the screenhouse samples, two vs. three canes for the field samples) (Figure 6A,B). The results indicate that growing conditions accounted for approximatively 54% (*p* < 0.001) of the metabolite variability in must and 44% (*p* < 0.001) in wines (Figure 6A,B, respectively). The effect of the different viticultural practices accounted for 38% (*p* < 0.01) of the total metabolite variability in must samples and 30% (*p* < 0.001) in wine samples. In the screenhouse experiment, the experimental effect of WD was quantified as approximatively 11% (*p* < 0.001) and 8% (*p* < 0.001) of the metabolite variations in must and wines, respectively. Pruning type (not part of the original experiment, Figure S1) had a significant experimental effect on must and wine samples from both experiments, and in the field experiment, it was approximatively 6% (*p* < 0.001) and 4% (*p* < 0.01) in must and wine samples, respectively. In the screenhouse experiment, pruning type accounted for approximatively 23% (*p* < 0.001) of the metabolite variability in must and 6% (*p* < 0.001) in wines. Interestingly, the treatment- and pruning type-related variability (and effect size) is attenuated by the fermentation process (Figure 6A,B).

### 2.5. In-Depth Assessment of Metabolite Formation during Vinification

The chemical changes (as individual metabolites) occurring during the winemaking process, from must to wine, were analyzed and are described in the following sections. Metabolites identified and quantified in must are amino acids, sugars, and organic acids. Only metabolites whose concentrations significantly changed as result of different treatments are described for wines.

#### 2.5.1. Field Experiment

The baseline-resolved signals from 11 amino acids (i.e., alanine, arginine, glutamate, glutamine, isoleucine, leucine, phenylalanine, proline, tyrosine, tryptophan, and valine), six sugars (i.e., glucose, fructose, sucrose, arabinose, xylose and gentiobiose), and three important organic aids (i.e., lactate, succinate, and citrate) were quantified in all must samples. Metabolite fluctuations in must samples from the field experiment are shown in Figures S2, S4A and S5A as box and whiskers plots. As it can be observed in Figure S2, DEF alone significantly decreased the amount of most amino acids. Differently, CT treatments significantly increased the levels of proline and the aromatic amino acids, phenylalanine and tyrosine. The combined DCT treatment significantly increased the levels of branched-chain amino acids (BCAA—leucine, isoleucine, and valine) in must samples. In Figure S4A, the concentration fluctuations of glucose, fructose, sucrose, arabinose, xylose and gentiobiose are shown. As expected, both CT treatments significantly increased the concentration of sugars in the must samples, while DEF decreased it. The concentration fluctuations of citrate, lactate, and succinate in must from the field experiment are shown in Figure S5A. Overall, DEF decreased the concentration of all organic acids in must samples. DCT increased citrate, while all treatments decreased succinate in must when compared to the control samples. As for the wine samples (Figure S6A–C), primary alcohols methanol and propanol were highest in CT and lowest in DEF samples. Isopentanol was highest in wines that underwent DCT and lowest in wines from DEF vines. Tyrosol was found to be highest in DCT wines—no significant differences were observed in DEF and CT plants when compared to the control. Phenylethanol was lowest in DEF wines and highest in DCT, with CT and control wines having values in between the former two. 2,3-butanediol displayed a similar trend. Concerning the amino acid composition, methionine was found to be highest in CT plants and lowest in DEF plants, with DCT and control wines having values in between the former two. Proline was highest in both CT treatments and lowest in DEF wines. Valine was lowest in DEF. Similar to what was observed for amino acids, citrate and lactate were decreased by DEF and were highest in CT wines. Succinate was highest in both CT treatments, while butyrate was decreased by both DEF and DCT treatments.

#### 2.5.2. Screenhouse Experiment

In Figure S3, the boxplots showing the amino acid fluctuations in must samples from the screenhouse experiment are displayed. As it can be observed, early- and mid-stress significantly increased the amount of glutamine and glutamate in must—the same trend was observed for alanine and arginine but was not significant. In contrast, the concentration of phenylalanine and tryptophan was significantly decreased by early- and mid-stress—the same trend was observed for BCAA but was not significant. Overall, late-stress did not significantly affect the amino acid composition of must samples when compared to the control, except for tyrosine, which was found to be lowest in all stressed samples. No significant effect could be observed for sugar accumulation in must (Figure S4B). Concerning the organic acids (Figure S5B), no significant changes in the lactate concentration could be observed, independently of the treatment. Citrate was decreased by all treatments. Succinate was significantly decreased by water deficit at ripening (late-stress). As for the wines samples (Figure S7), the phenolic compounds caffeic acid and *p*-coumaric acid, the organic acids fumarate and lactate, glutamate, butyrate, and propanol were significantly affected by different water stress treatments. Amongst them, caffeic acid was highest in control and early- stressed samples, while it was decreased by mid- and late-stress. *P*-coumaric acid exhibited the opposite trend. Fumarate and lactate had opposite trends, with fumarate being lowest in wines from early- and mid-stressed plants, while it was the highest in wines from the same treatment lactate. Glutamate was highest in early- and mid-stressed plants.

## 3. Discussion

In this study, ^1^H NMR metabolomics was employed to investigate the impact of different viticultural practices, including defoliation (DEF), crop thinning (CT), and irrigation (WD) on the chemical composition of must and wine samples from the cool-climate grape variety Solaris. The metabolomics investigation presented here complements our previously published study [3] by describing (1) the baseline metabolome of must and wines as a function of different growing conditions, (2) the changes in the bulk metabolome of must and wine samples obtained from different viticultural treatments, and (3) the changes in the bulk metabolome of must and wine samples from vines that underwent water deficit.

### 3.1. The Metabolome of Must and Wines as a Function of Different Growing Conditions

An overview of the chemical changes occurring in must and wines from different growing conditions (field vs. screenhouse) were obtained using PCA, which revealed substantial metabolic differences between field- and screenhouse-grown samples. Concerning the must samples from field and screenhouse, the main metabolic difference was that the latter had higher concentrations of amino acids (Figure 4A). This is not surprising as nitrogen in the form of ammonium and nitrate is an important component of the bulk nutrients provided to the vines through the irrigation water. Amino acids in winemaking serve as nutrients for yeast during fermentation, and imbedded in proteins, they influence wine stability, especially in white wine. The nitrogen content of the fermentation substrate (must) has previously been linked to the production of higher alcohols, important aroma compounds formed in wines during fermentation [45]. For instance, BCAA are structurally similar and important precursors of the higher alcohols isobutanol (valine) and isopentanol (isoleucine/leucine) and are linked through the Ehrlich pathway. In the same way, the aromatic amino acids phenylalanine, tyrosine, and tryptophan are precursors of the aromatic alcohols phenylethanol, tyrosol, and tryptophol, respectively. The impact of plant nitrogen status on wine quality is complex and often contradictory depending on the main factors’ influence in a given study [45]. In contrast organic acids, citrate, succinate, and malate were found to be at higher concentrations in the field samples (Figure 4A). Malate is one of the prevalent acids in grapes. Unlike tartrate, the content of malic acid and citric acid is known to decline over the course of the growing season [46], which in this experiment occurred earlier in the screenhouse vines due to the higher temperatures [3]. Grapes grown in cool regions often contain higher concentrations of acids, which may result in tart wines. Malolactic fermentation can help reducing wine acidity through the bacterial conversion of malate into lactate, a milder acid. Citrate represents about 5% of the total acid content in grapes and like malate and succinate, it can be easily metabolized by wine microorganisms to form lactic acid or acetic acid. The concentration of tartaric acid as measured by ^1^H NMR showed inconsistent results with FT-IR measurements and thus has been excluded from all PCA models. The reason for the observed inconsistency can be ascribed to the fact that the buffer solution used for NMR sample preparation contained potassium phosphate salts, which may have altered the natural equilibrium between the three main potassium tartrate-related species present in wines, namely tartaric acid, hydrogen tartrate, and tartrate. Of these, hydrogen tartrate precipitates with potassium.

As for the wines, valine, glutamate, and proline were identified in all samples and at higher concentrations in the screenhouse wines (Figure 4B). They are normally associated with a specific mouthfeel: valine with a bitter taste, proline with a sweet taste, and glutamate with umami taste. Proline is not part of the yeast assimilable amino acids, but it plays a key role in the amino acid turnover as well as in the perception of wine taste [47,48,49]. In fact, our saliva proteins are rich in proline, which contribute to the astringency mouthfeel [49]. 

Caffeic acid and *p*-coumaric acid were tentatively assigned in the ^1^H NMR spectra of wines from both the field and screenhouse experiment. It is important to stress that phenols in wine can exist both in the glycosylated and free form—the latter being formed during the winemaking process [8]. The two forms are difficult to distinguish by 1D ^1^H NMR. Overall, phenolic compounds were found to be slightly higher in the wines from the field experiment. This may be related to a general higher physiological maturity in the slightly warmer screenhouse compared to the field, as the phenolic concentration tend to decrease with increasing maturity [50]. 

### 3.2. Metabolic Fluctuations as a Function of Defoliation and Crop Load Reduction

The amino acid composition of must samples obtained from the field experiment was significantly affected by the different viticultural practices. Overall, when compared to the *control* samples, DEF decreased the amino acid concentration, while CT treatments significantly increased the concentration of phenylalanine, tyrosine, BCAA, and proline (Figure S2). The same trend could be observed for the sugars, which were found in highest concentrations in must from CT and DCT vines (Figure S4A). Similarly to sugar accumulation, proline accumulation in grapes has often been used as a marker of berry maturity [51]. In plants, proline covers numerous roles including osmotic adjustment, detoxification of ROS, and protection of membrane integrity [52]. Proline can control its own synthesis, and in developing berries, it can be synthesized starting from glutamate and/or glutamine. It has been demonstrated that Δ^1^-pyrroline-5-carboxylate synthetase, a key regulatory enzyme required for the synthesis of proline from glutamate, is present in grape berries throughout their development [48]. In wines, CT and DCT led to the highest levels of alcohols including ethanol, methanol, propanol, and isopentanol (Figure S6A). Monohydroxylic alcohols are formed by yeast and their synthesis depends on the chemical composition of must, yeast strain, and fermentation conditions [47]. Higher alcohols, which are normally present in wines at the concentration of 0.3 and 0.5 g/L, influence the organoleptic characteristics of wine. In particular, isopentanol has a sharp burning taste and has been associated with banana flavor. As for methanol, CT treatments led to the highest ethanol and glycerol concentrations (Table S2). While methanol is normally produced from grape skin pectins by endogenous pectinase enzymes, ethanol and glycerol are primary products of alcoholic fermentation, both significantly contributing to the final wine flavor in terms of sweetness (glycerol) and pungent taste (ethanol). Succinate was also found to be highest in CT plants (Figure S6C). This is in agreement with literature data where succinate has been shown to follow the time-course evolution of ethanol during alcoholic fermentation [39]. The combined DCT treatment led to the highest tyrosol content in field wines. Since all wines were made using the same yeast cultures, the observed fluctuations in the tyrosol concentration are likely to be ascribed solely to the viticultural practices. Tyrosol is a phenolic compound present in red and white wines that has been shown to be able to exert strong antioxidant activity in in-vitro studies [53]. 

### 3.3. Metabolic Fluctuations as a Function of Water Deficit

Global warming has led to the development of new viticultural areas in Northern Europe [54]. Even though water availability is normally not an issue in Northern European countries, unusually warm growing seasons and periodically scarce rainfall have significantly increased the risk of drought in these regions. This has raised the concern as to whether even short periods of drought could affect the chemical composition of wines. The present study has examined for the first time the effect of water deficit on the chemical composition of the cool-climate grape variety Solaris. PCA performed individually on the must and wine metabolite concentrations from the screenhouse experiment revealed that water deficit at pre-veraison and veraison has a higher impact on must and wines chemistry than water deficit during ripening (Figure 5C,D). In particular, early- and mid-stress significantly increased the concentration of glutamine and glutamate in must, which are related to umami/savory taste in wine (Figure S3). On the other hand, early and mid-stress decreased the amount of phenylalanine and tyrosine, which are precursors of the secondary aroma compounds phenylethanol and tyrosol in wines (Figure S3). As for the wines, malic acid was significantly lowered by early- and mid-stress (Table S1), while glutamate was highest in wines from early- and mid-stressed plants (Figure S7). Concerning phenolic compounds, caffeic acid and *p*-coumaric acid displayed opposite trends, with caffeic acid being highest in control and early-stress wines. Caffeic acid and *p*-coumaric acid are derivative of cinnamic acid, which in wines mainly derive from the stems, seeds, and skins and are extracted during the maceration period of winemaking [55]. No significant changes were observed for ethanol, glycerol, and acetic acid, primary flavor compounds in wines (Table S1). 

### 3.4. Concluding Remarks

The present multidisciplinary study aimed at investigating the impact of water deficit, defoliation, and crop thinning on Solaris’ plant and fruit development (Part 1) as well as on the bulk metabolic composition of Solaris must and wines as measured by FT-IR and ^1^H NMR (Part 2). Overall, our results show that, from an agronomical point of view, Solaris has a remarkable ability to tolerate and recover from water stress [3]. Nevertheless, short-term water deficit can significantly affect the concentration of several flavor-related compounds, including glutamate, butyrate, and propanol, of the organic acids lactate and fumarate, and of the phenolic compounds caffeic acid and *p*-coumaric acid. Growing conditions (field vs. screenhouse) as well as viticultural practices (defoliation and crop thinning) were found to be the main factors affecting the quality/chemical composition of Solaris must and wines. While the main chemical differences between the must and wine control samples from the field and screenhouse are mainly related to the nutrient-enriched irrigation system in the controlled screenhouse set up, defoliation and crop thinning showed a major impact on the chemical composition of Solaris wines—mainly in terms of compounds normally related to fruity aroma (i.e., isopentanol), non-sugar sweetness (i.e., proline and glycerol), and alcohol content. Tyrosol, which might be attractive as a natural phenolic antioxidant with high bioavailability, was increased in wines by a combination of defoliation and crop thinning. 

In conclusion, our preliminary results show that, although wine preference is subjective, wine quality—in terms of flavor chemistry—can be optimized by fine-tuning viticultural practices and the winemaking process. It must be stressed that our results, although promising, are based on a one-year trial (vintage) and further experiments are required to confirm these findings.

## 4. Materials and Methods

### 4.1. Experimental Design

Experiments were carried out in 2018 at the Pometum (Taastrup, Denmark), the experimental orchard of the University of Copenhagen, and consisted of two independent trials. Both studies aimed at investigating the chemical changes occurring in must and wine samples as a result of (1) water deficit (screenhouse experiment) and (2) different viticultural practices, namely defoliation (DEF) and crop thinning (CT) (field experiment). The water deficit experiment was carried out in a screenhouse (Figure 1) where 36 pot-grown vines underwent water deficit at three different phenological stages of berry development, namely pre-veraison (early-stress), veraison (mid-stress), and ripening (late-stress). The field experiment was performed in a north-south oriented vineyard where 32 vines underwent different viticultural treatments, namely crop thinning (CT), defoliation (DEF), and a combination of the above (DCT). Chemical changes in must and wine samples were measured by both FT-IR spectroscopy (WineScan) and ^1^H NMR spectroscopy. A more detailed description of the experimental design is given in Figure 1 and Part I [3].

### 4.2. The Winemaking Process

A detailed description of must sample preparation and collection is given in Part I [3]. As for the wines, after pressing, the must was racked into 2 L glass jars, inoculated with commercial yeast *Saccharomyces cerevisiae bayanus* (Lalvin DV10TM, Lallemand, Denmark), and moved to the fermentation room set to 17 °C. Fermentation was monitored via density and temperature control. After fermentation, 5% sulphite solution was added to the wines (1.5 mL/L equal to 75 mg sulphite/L), which was then transferred to a room set to 3 °C for 1 week of cold settling. Subsequently, samples were analyzed by FT-IR, and a second aliquot frozen at −80 °C for future NMR analysis.

### 4.3. FT-IR Analysis

Samples were analyzed by FT-IR spectroscopy using the WineScan instrument (WineScan FT 120, FOSS A/S, Hillerød, Denmark). More instrument specifications can be found in Part I [3]. Oenological parameters monitored in the present study are ethanol content (%vol), malic acid (g/L), pH, tartaric acid (g/L) total acidity (g/L), glycerol (g/L), volatile acidity (g/L), fructose (g/L), and reducing sugar (g/L) (see Tables S1 and S2).

### 4.4. NMR Analysis

#### 4.4.1. Chemicals

Deuterium oxide (D_2_O, 99.9%), potassium phosphate monobasic (KH_2_PO_4_), and sodium 3-trimethylsilyl-propionate-2,2,3,3-d4 (TSP) were purchased from Sigma-Aldrich (Darmstadt, Germany).

#### 4.4.2. Sample Preparation

For each sample, a total of 3 mL was centrifuged (Scanspeed 1580 R, Labogene, Denmark) at 2000 rpm at 4 °C for 30 min to precipitate residual solids. Sample preparation for must and wine analysis by ^1^H NMR was performed as described by Aru et al. [56]. Briefly, an aliquot of 700 µL of sample was transferred to a 1 mL Eppendorf tube containing 300 µL of 1 M phosphate buffer (pH = 4.5) with D_2_O (20%) and TSP (5 mM). To ensure homogeneity while avoiding foaming, each sample was gently vortexed for 1 min. For each sample, 600 µL were transferred into a 5 mm O.D. NMR SampleJet tube (Bruker BioSpin, Ettlingen, Germany).

#### 4.4.3. NMR Measurements

NMR spectra were recorded on a Bruker Avance III 600 operating at a proton Larmor’s frequency of 600.13 MHz and equipped with a 5 mm broadband inverse (BBI) probe (Bruker BioSpin, Ettlingen, Germany). Data acquisition and processing were carried out using TOPSPIN3.6. After temperature equilibration (5 min), ^1^H-NMR spectra were measured at 298 K using the standard pulse sequence for presaturation of the water signal (*zgcppr* pulse program, Bruker nomenclature), a sweep width of 12,626 Hz (21 ppm), a 90◦ pulse, and an acquisition time of 3 s. The relaxation delay was set to 4 s. Spectral data were collected into 64K data points, after 256 scans. The receiver gain was fixed for all the experiments to an adequate value estimated through several tests for the juice and wine samples. NMR spectra were acquired in automation using IconNMR and the SampleJet system (Bruker BioSpin, Ettlingen, Germany). Phase and baseline correction were performed in TOPSPIN. 

#### 4.4.4. Data Pre-Processing and Multivariate Analysis

NMR data for must and wines were imported separately into MATLAB 2020b (Mathworks Inc., Natick, MA, USA) where the ^1^H NMR spectra were referenced to the TSP singlet at 0.00 ppm and signals aligned using the icoshift algorithm [57]. A total of 30 resonances were quantified in the must and 49 resonances in wines. Metabolite assignments (level 2) are based on literature data (J-coupling and chemical shift) [34,39,41,42] and the yeast metabolome database (http://www.ymdb.ca/; accessed on 15 April 2022). Baseline-resolved signals were quantified (relative concentration) by raw sum of spectral intensities. The datasets of metabolite concentrations were imported into the PLS_Toolbox (version 7.5.1, Eigenvector Research, Manson, WA, USA) where the metabolites concentrations were autoscaled and analyzed by principal component analysis (PCA) [43] and ANOVA simultaneous component analysis (ASCA) [44] (1000 permutations). 

## Figures and Tables

**Table 1:** Studies on Solaris grape and wines per year 2000–2022. Web of Science was used to search for publications using “Solaris”, “grape”, and “wine” as topic keywords.

Publication title	Year	Reference
Different susceptibility of European grapevine cultivars for downy mildew	2008	[1]
Adaptation to climate change: viniculture and tourism at the Baltic coast	2011	[4]
Instrumental and sensory characterisation of Solaris white wines in Denmark	2015	[5]
Influence of pre-fermentation treatments on wine volatile and sensory profile of the new disease tolerant cultivar Solaris	2015	[6]
Sequence analysis of loci Rpv10 and Rpv3 for resistance against grapevine downy mildew (*Plasmopara viticola*)	2015	[2]
The Nordic light terroir	2016	[7]
Targeted and untargeted high-resolution mass approach for a putative profiling of glycosylated simple phenols in hybrid grapes	2017	[8]
Impact of sequential co-culture fermentations on flavour characters of Solaris wines	2017	[9]
Phenolic compounds and antioxidant activity of twelve grape cultivars measured by chemical and electrochemical methods	2018	[10]
Shikimic acid concentration in white wines produced with different processing protocols from fungus-resistant grapes growing in the Alps	2018	[11]
Evaluation of mechanical properties of berries on resistant or tolerant varieties of grapevine	2019	[12]
Use of the NeoViGen96 chip to understand the defense status of cultivars and resistant genotypes of *Vitis vinifera*	2019	[13]
Occurrence of Ehrlich-derived and varietal polyfunctional thiols in Belgian white wines made from Chardonnay and Solaris grapes	2020	[14]
Exploring the typicality, sensory space, and chemical composition of Swedish Solaris wines	2020	[15]
Press fractioning of grape juice: a first step to manage potential atypical aging development during winemaking	2020	[16]
Effects of water stress, defoliation and crop thinning on *Vitis vinifera* L. cv. Solaris. Part I: plant responses, fruit development and fruit quality	2022	[3]

## Data Availability

The data presented in this study are available on request from the corresponding author.

## Supplementary Materials

The following supporting information can be downloaded at: https://www.mdpi.com/article/10.3390/metabo12070672/s1. Table S1: Results from the FT-IR (WineScan) analysis of the wine samples from the screenhouse trial. Control = no treatment, early-stress = after flowering, mid-stress = before veraison, late-stress = during ripening. ANOVA was used to assess the variation between different groups. Different letters stand for statistically significant differences between the groups (*p* < 0.05); ns = not significant. (* calculated as tartaric acid equivalents). Table S2: Results from the WineScan analysis of the wine samples from the field trial. Control = no treatment; DEF = defoliation; CT = crop thinning; DCT = defoliation and crop thinning. ANOVA was used to assess the variation between different groups. Different letters stand for statistically significant differences between the groups (*p* < 0.05); ns = not significant. (* calculated as tartaric acid equivalents). Figure S1A–D: Biplots of the PCAs performed on the metabolite concentrations of must and wine samples from the field experiment (A and B, respectively), and must and wine samples from the screenhouse experiment (C and D, respectively). Samples are colored based on the pruning type. Keys: Ace: acetate; Ala: alanine; Ara: arabinose; Arg: arginine; BDO: 2,3-butanediol; But: butyrate; Caf: caffeic acid; Chol: choline; Cit: citrate; EtOH: ethanol; Fru: fructose; Glc: glucose; Gln: glutamine; Glu: glutamate; Glyc: glycerol; IamOH: isoamyl alcohol; IbuOH: isobutanol; Ile: isoleucine; Lac: lactate; Leu: leucine; Mal: malate; MeOH: methanol; pCoum: *p*-coumaric acid; PheOH: phenylethanol; Pro: proline; TyrOH: tyrosol; Suc: succinate; Val: valine; Xyl: xylose. Figure S2: Box and whiskers plots showing the amino acid distribution in must samples from the field experiment. Keys: FC=field control; DEF=defoliation; CT=crop thinning; DEF-CT= defoliation and crop thinning. Vertical lines indicate the span of the distributions, the middle line represents the median value of the observations, and the red dot represents the mean value of the observations. Filled circles represent outliers (values > 1.5 × IQR). ANOVA was performed to assess the statistical significance of the results. Different letters stand for significant differences between the groups. Figure S3: Box and whiskers plots showing the amino acid distribution in must samples from the screenhouse experiment. Keys: HC= screenhouse control; ES= early-stress; MS= mid-stress; LS= late-stress. Vertical lines indicate the span of the distributions, the middle line represents the median value of the observations, and the red dot represents the mean value of the observations. Filled circles represent outliers (values > 1.5 × IQR). ANOVA was performed to assess the statistical significance of the results. Different letters stand for significant differences between the groups. Figure S4: Box and whiskers plots showing the sugar distribution in must samples from the field (A) and screenhouse (B) experiments. See captions to Figure S2 (field) and S3 (screenhouse) for legend and keys explanation. Figure S5: Box and whiskers plots showing the distribution of organic acids in must samples from the field (A) and screenhouse (B) experiments. See captions to Figure S2 (field) and S3 (screenhouse) for legend and keys explanation. Figure S6: Box and whiskers plots showing the distribution of alcohols (A), amino acids (B) and organic acids (C) identified and quantified in wines from the field experiment whose concentration significantly changed amongst different treatments. See caption to Figure S2 for legend and keys explanation. Figure S7: Box and whiskers plots showing the distribution of metabolites identified and quantified in wines from the screenhouse experiment whose concentration significantly changed amongst different water deficit treatments. See caption to Figure S3 for legend and keys explanation.
