# Supplementary material for: Effects of Water Stress, Defoliation and Crop Thinning on Vitis vinifera L. cv. Solaris Must and Wine Part II: 1H NMR Metabolomics
Source: Metabolites. 2022 Jul 21;12(7):672. doi: 10.3390/metabo12070672 (PMC9316113; doi:10.3390/metabo12070672)
Supplement: Supplementary file 1 [file metabolites-12-00672-s001.zip › metabolites-1765275-supplementary.pdf]

Supplementary information

Effects of water stress, defoliation and crop thinning on *Vitis vinifera* L. cv. Solaris must and wine: Part II: ^{1}H NMR metabolomics

Violetta Aru [1†*], Andreas Paul Nittnaus [2,3†], Klavs Martin Sørensen[1], Torben Bo Toldam-Andersen[2] and Søren Balling Engelsen[1*]

[1]Department of Food Science, University of Copenhagen, Rolighedsvej 26, DK-1958 Frederiksberg, Denmark; violetta@food.ku.dk (V.A.); kms@food.ku.dk (K.M.S.); se@food.ku.dk (S.B.E.)

[2]Department of Plant and Environmental Sciences, University of Copenhagen, Højbakkegård Alle 13, DK-2630 Taastrup, Denmark; a.nittnaus@gmx.at (A.P.N.); tbta@plen.ku.dk (T.B.T.-A.)

[3]Instituto Superior de Agronomia, Universidade de Lisboa, Tapada da Ajuda, 1349-017 Lisboa, Portugal

[*]Correspondence: violetta@food.ku.dk (V.A.); se@food.ku.dk (S.B.E.)

[†]These authors contributed equally to this work.

Results

1. FT-IR spectroscopy

Table S1. Results from the FT-IR (WineScan) analysis of the wine samples from the screenhouse trial. Control = no treatment, early-stress = after flowering, mid-stress = before veraison, late-stress = during ripening. ANOVA was used to assess the variation between different groups. Different letters stand for statistically significant differences between the groups (p<0.05); ns = not significant. (* calculated as tartaric acid equivalents).

Parameters	Control	Early-Stress	Mid-Stress	Late-Stress	p-value
**Ethanol, %vol**	13.42	13.34	13.03	12.92	ns
**Malic acid, g/l**	2.01^{b}	1.77^{a}	1.77^{a}	1.82^{ab}	<0.05
**pH**	3.15	3.16	3.13	3.13	ns
**Tartaric acid, g/l**	3.19	3.21	3.37	3.41	ns
**Total acidity, g/l***	7.85	7.73	7.79	7.82	ns
**Glycerol, g/l**	7.31	7.21	7.08	6.97	ns
**Volatile acidity, g/l**	0.13	0.16	0.14	0.12	ns
**Fructose, g/l**	1.23	1.00	1.06	1.03	ns
**Reducing sugar, g/l**	0.57	0.28	0.51	0.42	ns

Table S2. Results from the WineScan analysis of the wine samples from the field trial. Control = no treatment; DEF = defoliation; CT = crop thinning; DCT = defoliation and crop thinning. ANOVA was used to assess the variation between different groups. Different letters stand for statistically significant differences between the groups (p<0.05); ns = not significant. (* calculated as tartaric acid equivalents).

Parameters	Control	DEF	CT	DCT	*p-value*
**Ethanol, %vol**	13.27^{b}	12.52^{a}	14.44^{c}	14.3^{c}	<0.05
**Malic acid, g/l**	3.63^{ab}	3.58^{ab}	3.43^{a}	3.73^{b}	<0.05
**pH**	3.01^{b}	2.88^{a}	3.11^{c}	3.04^{bc}	<0.05
**Tartaric acid, g/l**	4.17^{ab}	4.87^{b}	2.64^{a}	3.19^{ab}	<0.05
**Total acidity, g/l***	9.68^{b}	10.24^{b}	8.93^{a}	9.66^{b}	<0.05
**Glycerol, g/l**	7.48^{b}	6.23^{a}	8.39^{c}	7.88^{b}	<0.05
**Volatile acidity, g/l**	0.23^{a}	0.23^{a}	0.28^{b}	0.24^{a}	<0.05
**Fructose, g/l**	1.17	1.19	1.73	1.78	ns
**Reducing sugar, g/l**	0.76	0.86	1.46	1.47	ns

2. Metabolomics analysis of must and wine samples from the field and screenhouse experiments – pruning type-related variability

In order to scrutinize for possible additional sources of variability that potentially can be related to the observed high inter-sample diversity, PCA scores in Figure 5A-D (main manuscript) were colored based on the pruning type (one vs. two canes - screenhouse samples, and two vs. three canes - field samples). The results are shown in Figure S1A-D. Pruning type is a major contributor to the metabolite variability in both must and wine samples from both growing conditions (field and screenhouse) – for effect size see Figure 6 in main text. In general, the lower the number of canes, the higher is the metabolite concentrations in the must and wine samples.

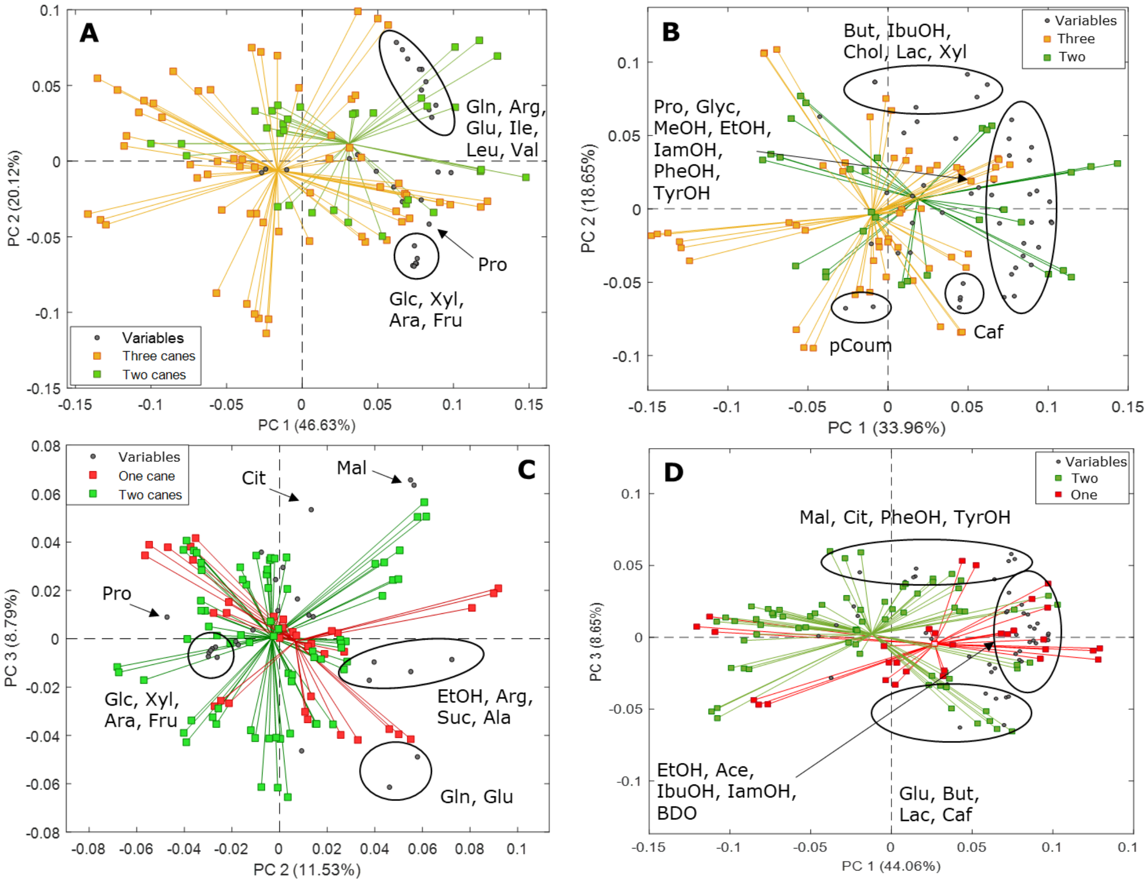

Figure S1A-D. Biplots of the PCA performed on the metabolite concentrations of must and wine samples from the field experiment (A and B, respectively), and must and wine samples from the screenhouse experiment (C and D, respectively). Samples are colored based on the pruning type. Keys: Ace: acetate; Ala: alanine; Ara: arabinose; Arg: arginine; BDO: 2,3-butanediol; But: butyrate; Caf: caffeic acid; Chol: choline; Cit: citrate; EtOH: ethanol; Fru: fructose; Glc: glucose; Gln: glutamine; Glu: glutamate; Glyc: glycerol; IamOH: isoamyl alcohol; IbuOH: isobutanol; Ile: isoleucine; Lac: lactate; Leu: leucine; Mal: malate; MeOH: methanol; *p*Coum: p-coumaric acid; PheOH: phenylethanol; Pro: proline; TyrOH: tyrosol; Suc: succinate; Val: valine; Xyl: xylose.

3. In depth assessment of metabolite formation during vinification

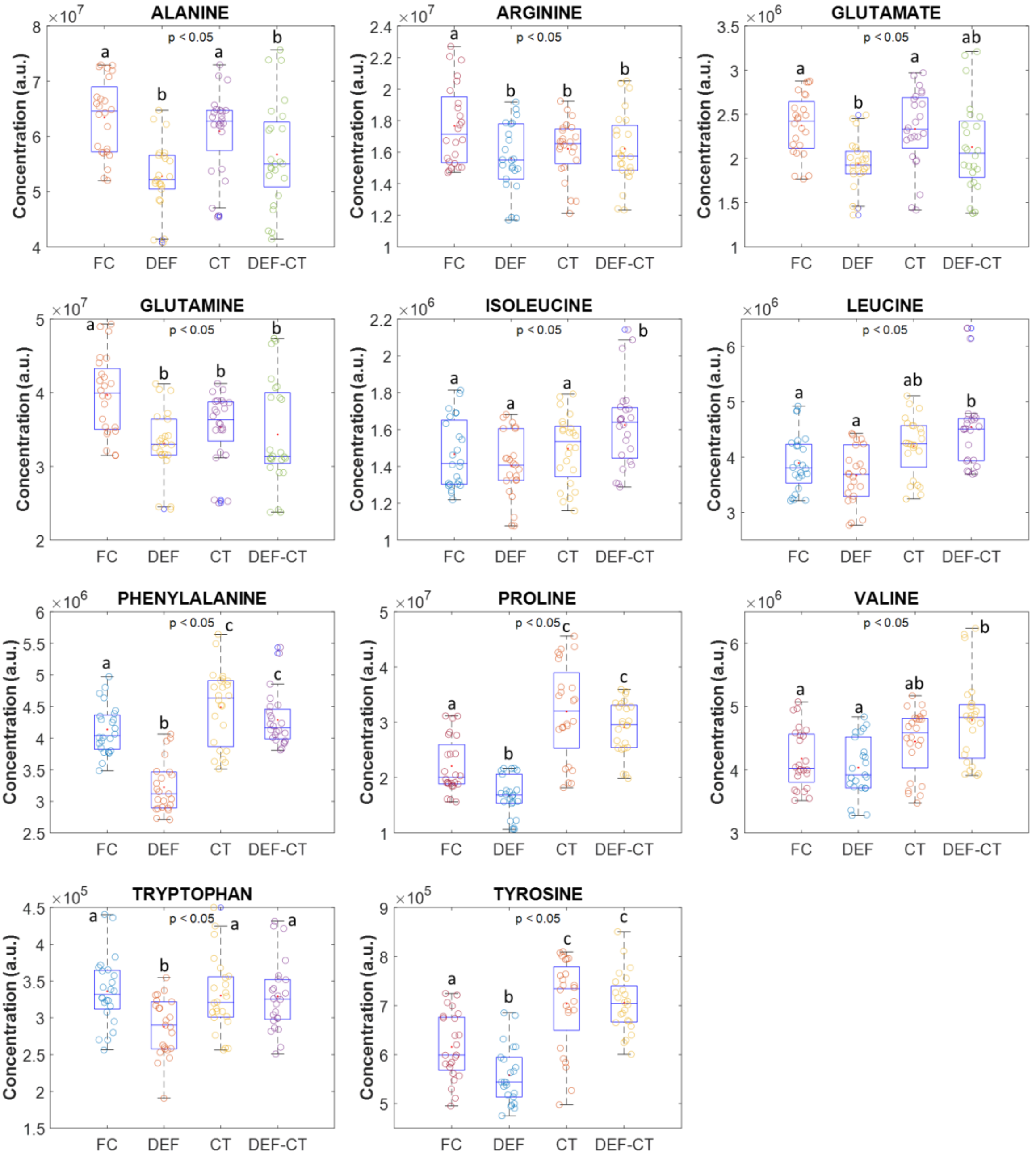

Figure S2. Box and whiskers plots showing the amino acid distribution in must samples from the field experiment. Keys: FC=field control; DEF=defoliation; CT=crop thinning; DEF-CT= defoliation and crop thinning. Vertical lines indicate the span of the distributions, the middle line represents the median value of the observations, and the red dot represents the mean value of the observations. Filled circles represent outliers (values > 1.5 × IQR). ANOVA was performed to assess the statistical significance of the results. Different letters stand for significant differences between the groups.

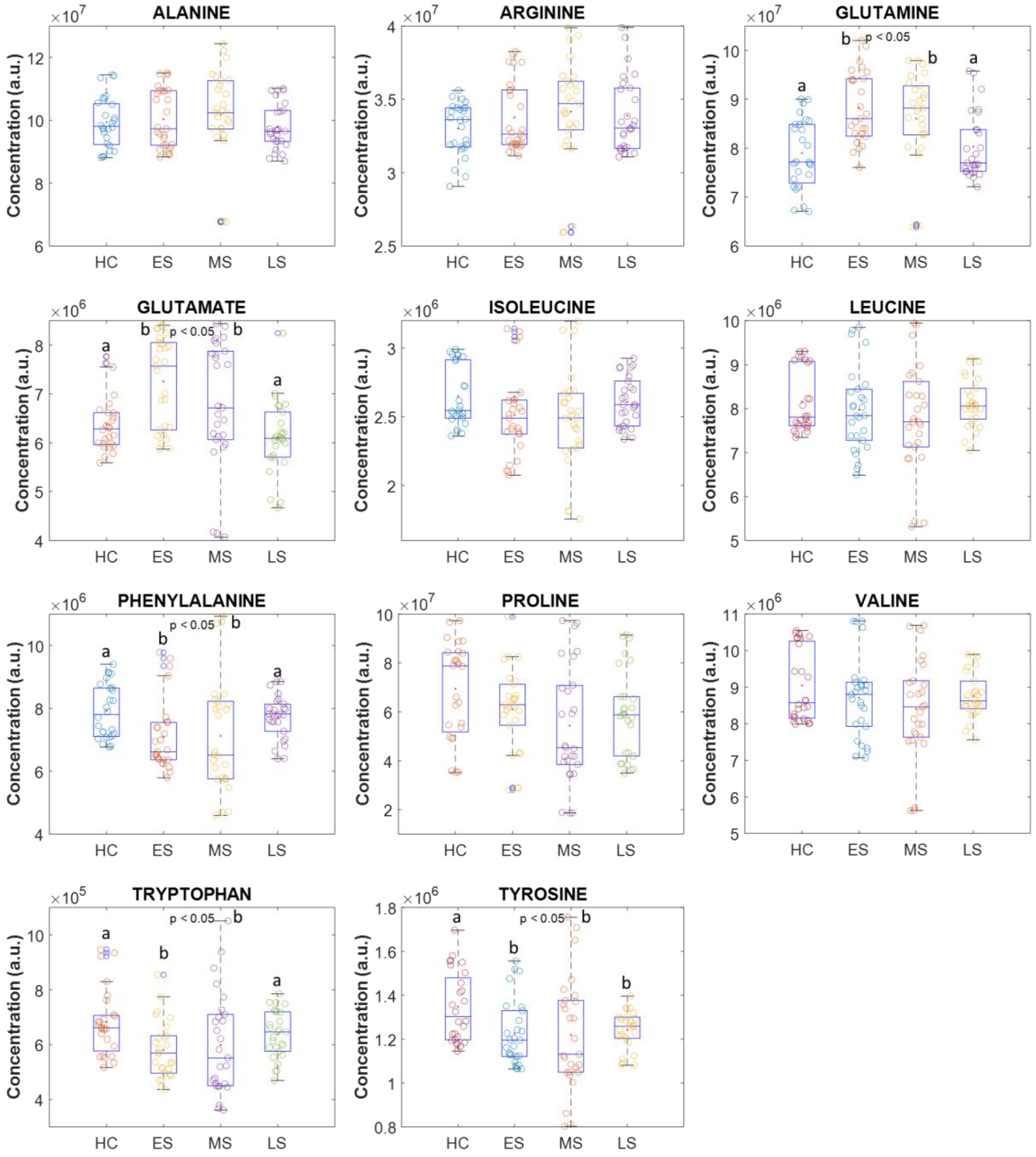

Figure S3. Box and whiskers plots showing the amino acid distribution in must samples from the screenhouse experiment. Keys: HC= screenhouse control; ES= early-stress; MS= mid-stress; LS= late-stress. Vertical lines indicate the span of the distributions, the middle line represents the median value of the observations, and the red dot represents the mean value of the observations. Filled circles represent outliers (values > 1.5 × IQR). ANOVA was performed to assess the statistical significance of the results. Different letters stand for significant differences between the groups.

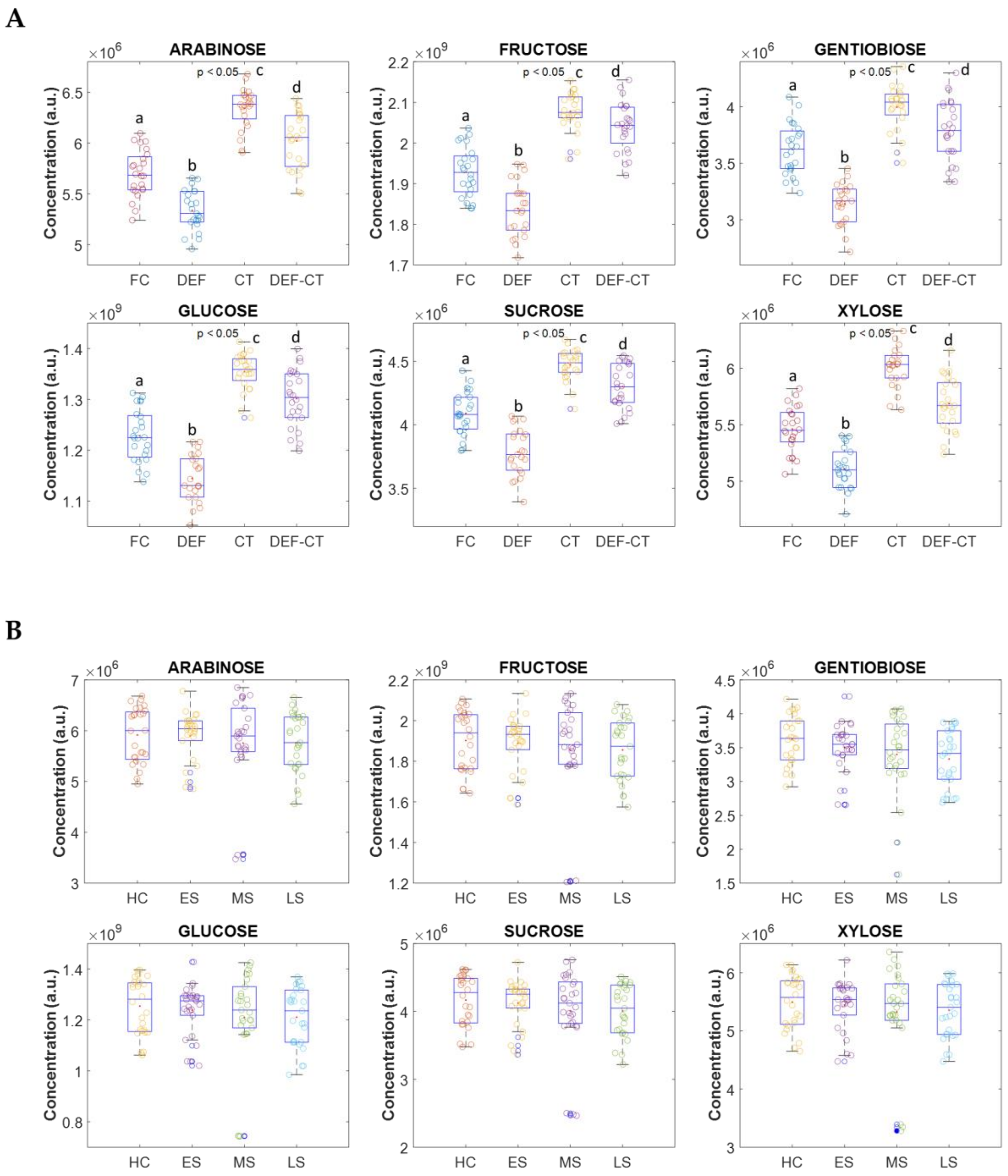

Figure S4. Box and whiskers plots showing the sugar distribution in must samples from the field (A) and screenhouse (B) experiments. See captions to Fig. S2 (field) and S3 (screenhouse) for legend and keys explanation.

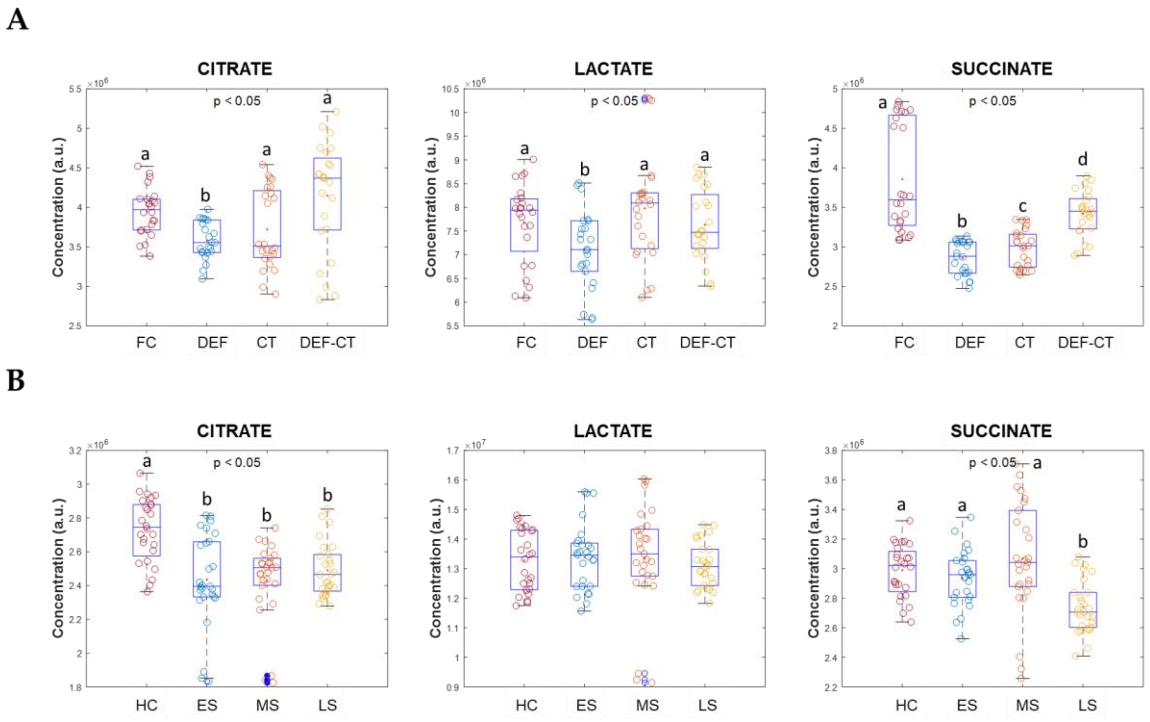

Figure S5. Box and whiskers plots showing the distribution of organic acids in must samples from the field (A) and screenhouse (B) experiments. See captions to Fig. S2 (field) and S3 (screenhouse) for legend and keys explanation.

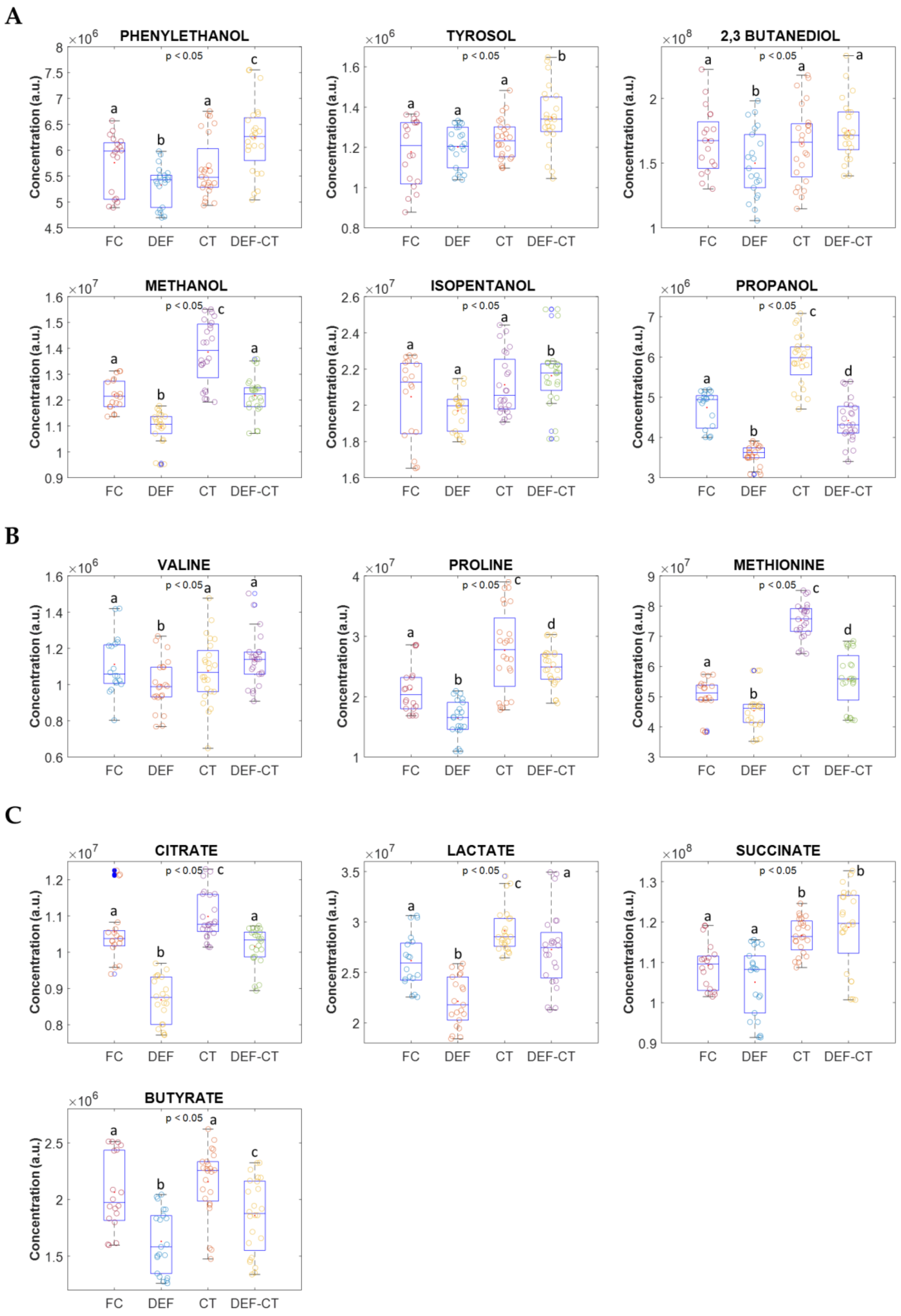

Figure S6. Box and whiskers plots showing the distribution of alcohols (A), amino acids (B) and organic acids (C) identified and quantified in wines from the field experiment whose concentration significantly changed amongst different treatments. See caption to Fig. S2 for legend and keys explanation.

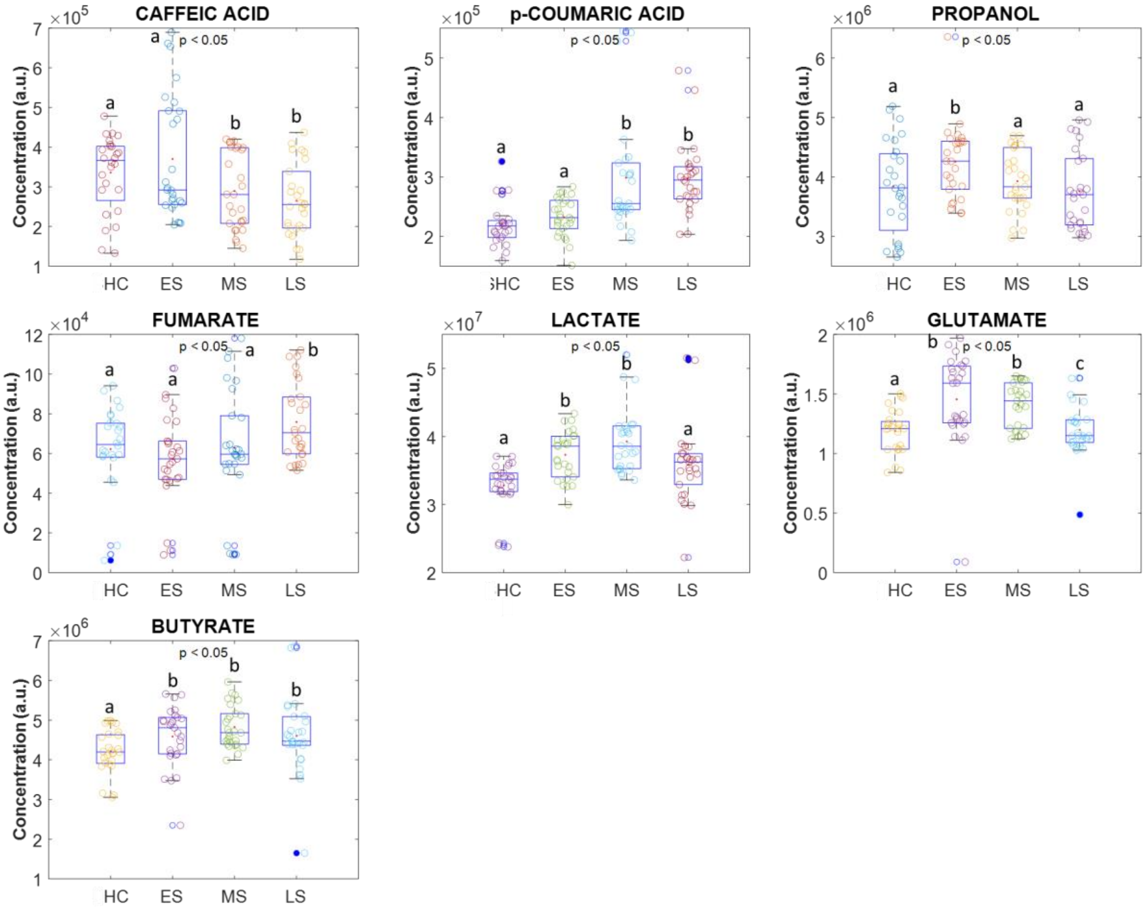

Figure S7. Box and whiskers plots showing the distribution of metabolites identified and quantified in wines from the screenhouse experiment whose concentration significantly changed amongst different water deficit treatments. See caption to Fig. S3 for legend and keys explanation.
